# Making waves: A vision for digital water utilities

**DOI:** 10.1016/j.wroa.2023.100170

**Published:** 2023-02-04

**Authors:** Magnus Arnell, Maya Miltell, Gustaf Olsson

**Affiliations:** a Division of Industrial Electrical Engineering and Automation (IEA), Department of Biomedical Engineering, Lund University, PO Box 118, Lund SE-22100, Sweden; b RISE Research Institutes of Sweden, PO Box 857, Borås 501 15, Sweden

**Keywords:** Digitalization, Utility management, Data management, Change management

## Abstract

Digitalisation has developed over half a century and is one of the global trends defining society of today and future. Digitalisation is envisioned to help water utilities to become: i) community orientated and digitally integrated with customers and society; ii) digitally transformed end-to-end throughout the value-chain and interconnected between business units; iii) predictive & proactive, utilizing models and applications for control and decision support; iv) visually communicative with customers and society, creating customers aware of the value of water; and financially sustainable by optimal operation (OPEX), and sustainable investments (CAPEX). Digitalisation is a process for business development, where digital solutions are used for automation and innovation. Utilizing the potential of the technological innovation requires a parallel organisational transformation. Any implementation of systems or applications must be motivated in actual needs for the organisation and service delivery. Prior to any digitalisation, identifying issues and areas of improvement is essential. Starting the digital journey, motivating employees, improving the digital culture and creating acceptance of new processes are needed on all levels. Most digital applications require collection, storage, sharing and integrated analysis of large amounts of data. This includes both soft and hard digital infrastructure.

## Highlights

• Water utilities are envisioned to be community orientated, digitally transformed.
• Furthermore, to be proactive, visually communicative and financially sustainable.
• Digitalisation based in organisational needs and improvement of services.
• Organisational and technical developments are needed for digitalisation.
• Both soft and technical digital infrastructure is required for interoperability.

## Graphical abstract

**Figure:**
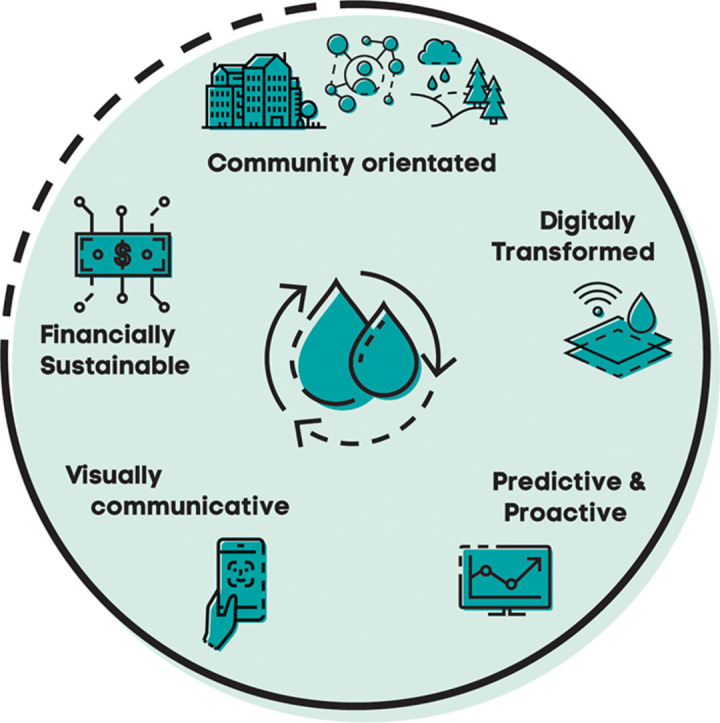

## 1 Introduction

Digitalisation is one of the global trends defining society today and for the future. It is highly relevant in all industrial sectors due to increased pressures to meet societal demands on efficiency and sustainability (Xu et al., 2018). Water utilities face increasing expectations on services while water resources diminish and deteriorate as a result of climate change (Earman and Dettinger, 2011; Heidari et al., 2021; Hongve et al., 2004; Lettenmaier et al., 1999). Adopting circular systems, facing ageing infrastructure, demographic changes, and increased pressure from climate change create significant challenges (Arup, 2015; Cosgrove and Loucks, 2015; Ofwat, 2020). An integrated approach to digital transformation within water services is needed to enhance resource efficiency and meet new demands. While digitalization has global impacts the challenges and conditions vary from high to low-income countries. Institutional capacity and financing for transition are more challenging in developing countries, but there is less technical legacy to consider. Generally, a vision is needed to enthuse the water sector for the potential of digitalization and focus on value creation rather than on technical innovation.

The presented work has been developed in a 2-year project by a consortium of 23 utilities, companies and academic organisations in Sweden. The methods were literature review; collecting examples and experiences; a series of three workshops on potential value creation, capacity and knowledge; and cybersecurity. This article outlines vision and potential with digitalisation as well as main barriers for the transformation.

## 2 Digital transformation

Digitalisation is a continuous process and has been developing since half a century. The technical push has been revolutionary. While a typical computer memory was of the order kilobytes in the early 1970s we now can storage terabytes. Computing power is almost ‘free’ and communication technology allows huge information flows, for example demonstrated in internet of things (IoT) and artificial intelligence (AI). A prerequisite for the success of digitalisation is the sensor and instrumentation development, as well as the connectivity and the will to digitalise. The demand pull, including regulatory requirements, economics and efficiency have been key driving forces for the technology development. Digitalisation is a process for business development, where digital solutions are used for automation and innovation. This is not to be interchanged with ‘digitisation’ which simply refers to the making of something analogue into digital and does not entail the usage or culture. Digitalisation ought to be considered a means to uphold and innovate the services provided, increase business opportunities and connect to other municipal services. It is an ongoing journey.

Utilizing the potential of the technological innovation requires a parallel organisational transformation (Hamilton et al., 2021; Wang et al., 2021). The transition needs to be anchored in the top of the organisation to clearly point out a direction and allocate resources by defining roadmaps and strategies and by forming new key roles with distinct responsibilities (Grievson et al., 2022; Hamilton et al., 2021; SWAN Forum, 2019). However, this must involve the whole organisation (Vitasovic et al., 2022). Any implementation of gadgets or applications must be motivated by actual needs and have clearly stated objectives and expected impacts (e.g. on operation or services). Prior to any digitalisation, identifying issues and areas of improvement is essential. Starting the digital journey, motivating employees, improving the digital culture and creating acceptance of new processes will be needed on all levels (Ng et al., 2020; Taneja, 2018; Wahlström et al., 2020). Involvement in innovation initiatives such as pilots and tests can create valuable collaborations, understanding for potentials and act as accelerators for required development (Grievson et al., 2022). However, each pilot or project must be relevant and motivated for the organisation.

### 2.1 Potential value creation

Digitalisation holds no value in itself, but the targeted improvement of processes or services needs to be identified (Parviainen et al., 2017). Economic incentives such as ROI (return on investment) are commonly used, but other values such as brand recognition, environmental sustainability, customer experience, employee health and improved service are equally important (Gómez et al., 2017; Rahmani et al., 2022; Woodside and Sood, 2017). These can certainly have indirect economic benefits. Saint (2014) emphasizes that technical developments in smart cites need to benefit citizens to be successful. Digitalisation can contribute with information for better decision making, more efficient production processes and resource consumption, better use of personnel resources, and predictive maintenance, to name a few (Boyle et al., 2022; Grievson et al., 2022; Rahmani et al., 2022). Development in one area can create synergy effects in another sector. For example, installation of smart meters in real estate can create not only better services but also aid leakage detection. Therefore, short term ROI-analysis does not depict the true impact of a digital investment enabling long-term transformation (Giourka et al., 2019; Tkachenko et al., 2021).

Digitalisation has the potential to address issues and improve services throughout the utility. Ubiquitous monitoring, supervisory control, foresighted and integrated decision support and integration with external stake holders can be realized. However, it is crucial to analyse how measurements and information are going to be used and eventually to complete planned necessary integration throughout the data pipeline (Therrien et al., 2020). The applicability, sustainability and quality of the information must be ensured so that it can form a reliable basis for decisions, all the way from the operating level to the management level. By monitoring and control of interconnected systems, digital tools can provide opportunity for circular systems where water is differentiated in quality, correctly valued, wisely used, and where the contents of wastewater are viewed as resources (Arnell et al., 2021; D'Amico et al., 2022; Eggimann et al., 2017). Advanced use of modelling and prognostication, fault detection and status classification allow for better predictive maintenance and more effective investments (Chen et al., 2022). A better integrated organisation is possible where resources are efficiently utilized. Altogether, this also allows for a more sustainable economy for utilities.

### 2.2 Utility workforce and competences

As digitalisation in the future will penetrate all levels of the utility – systems and organisation – it is critical to identify how data and information are used at different levels of the organisation and how this affects the requirements.

» At the equipment operating level of treatment plants, automation systems and controllers require quality-checked measurements with a relatively high frequency (minutes and faster).
» Automatic controllers and operators rely on process measurements and observations. Monitoring, estimation, and control depend on models, process understanding, increasingly packaged as dynamic models or digital twins of various process parts. Typically, the time scale is from minutes to weeks.
» Plant managers and process engineers aim toward optimizing the operations, mostly requiring data at a wide range of time scales (hour to year) and wider scope of information, also beyond the fence of the plant in question.
» Plant maintenance should be based on updated equipment information, allowing adequate response times of maintenance needs. Temporal and spatial scales differ for strategic and tactic asset management (Marlow and Burn, 2008).
» Support functions like financial and economic management can mostly settle with long time scales (months to years) but business processes benefit from system wide integration of information.
» Utility management level mostly utilize information for decision support, aggregating and synthesizing information on averaged data for longer periods. Forecasting, prognostication and scenario analysis are key in decision support models.
» For visualization of information for customers, extensive and detailed information is presented in an aggregated and useful way.

In parts of the industrial sector the fourth industrial revolution is expected to lead to vast increase in productivity. Through automation and robotisation personnel resources are reduced (Davies, 2015). However, this does not necessarily hold universally. In economies where the degree of automation is low this potential still exists at production facilities, (e.g. water works and wastewater treatment plants). But, in most developed countries, basic automation and SCADA systems are already in place. While some repetitive tasks can still be removed, the operations staff has already been reduced from 24 to 7 operation and manual monitoring to on-line control and operators on standby duty outside business hours. In the administrative work of the industry, automation still has a large potential for removing repetitive tasks and saving time (Bossen and Ingemansson, 2016). This has recently been demonstrated by AI chatbots like ChatGPT by Open AI (Agrawal et al., 2022). This demonstrates the huge demand for increased knowledge of the pros and cons of AI.

In many applications automation may influence the design process to allow smaller safety margins, thus reducing capital cost. Obviously such a system is more sensitive to disturbances, putting a higher demand on competences and technical and organisational capabilities (Simic and Nedelko, 2019). Digitalisation will require new skills and new roles within utilities and their employees. New systems (hardware and software) will require new skills to operate and maintain. Areas of minor relevance today will grow in size and importance, e.g. instruments and sensors, data management, IT / OT infrastructure and cybersecurity (Hassanzadeh et al., 2020). Traditional water professionals will have to acquire new skills within the digital domain and utilities will have to recruit for new roles such as: digital manager, data scientist, programmer, systems engineer, cybersecurity expert etc. (Arnell et al., 2021; Simic and Nedelko, 2019).

## 3 Storing and sharing data as a requirement

Most digital applications require collection, storage, sharing and integrated analysis of large amounts of data, i.e. a digital infrastructure consisting of multiple components. This includes both soft and hard digital infrastructure. Soft digital infrastructure is the layer of for example organisational and legal requirements between the technical digital infrastructure and the business and services, Table 1 (Rudmark, 2022). Both existing systems in place and future needs must be considered. Cloud applications, wireless communication and IoT are enabling systems-interoperability and data sharing. Structured collection, storage and processing of data are fundamental for reaching the potential values. Sharing data internally and externally is critical for many high-level applications, e.g. decision support, prognostication and fusion and analysis of data (Carrico et al., 2020; Fernandez-Carames and Fraga-Lamas, 2019; SWAN Forum, 2019; Xu et al., 2018). To enable sharing of data to multiple applications, platforms and organisations, defined and standardized data models and interfaces for access are needed. Information classification and clear data ownership are necessary to securely share data and allow for use of different suppliers in the network of solutions, avoiding lock-in effects and inflexible systems (Barbero et al., 2018; Eggimann et al., 2017) . For water utilities, being part of the critical infrastructure, it is crucial to consider cyber- and information security (Fernandez-Carames and Fraga-Lamas, 2019; Rahmani et al., 2022; Tang and Meng, 2021). There was a common apprehension amongst the utilities in the study that on-premise storage in servers are more secure than outsourced cloud storage due to the increased amount of control. However, it is also associated with more responsibility to ensure security of the various communication layers, from the physical layer and upwards (Kim and Solomon, 2021).

**Table 1:** Examples of needs and infrastructure on organisational and technical level.

	Organisational	Technical
NEEDS	• Acceptance and digital culture • Clear responsibilities and processes defined, including data stewardship • Ability for O&M to influence digitalisation efforts and development • Budget allocation for development • Efforts and development anchored in clear needs and goals • Documentation of data models used and systems architecture • Support for technical procurement according to technical prerequisites	• Ability to collect, share and store data according to requirements and needs. ○ The organisation's needs ○ Data stream needs ○ Security requirements ○ Legislative requirements • Ability to translate, process and analyse data according to requirements and needs. • Flexibility in technical solutions - a key factor to be able to iterate systems and architecture to fit the organisation today and tomorrow.
INFRASTRUCTURE	Soft digital infrastructure • Standards • Data stewardship • Data models • Information classification • Organisational processes • Policies • Legal requirements	Technical digital infrastructure • IoT equipment and platforms • Communication networks and services • Cloud storage (outsourced or in-house) • On premise storage • SCADA and control systems

## 4 Vision for digital water utilities

Fulfilling the requirements and pursuing both the technical and organisational transformation paves the way to realize the potential values of digitalisation indicated in Section 2.1 and to achieve a vision for digital water utilities. The vision includes:

**Community orientated** and digitally integrated with customers and society. Data and information are shared between stakeholders (e.g. real estate companies, other utilities, municipal service organisations) and used for integrated planning and operation. New products and services are enabled, and new business models emerge. Water utilities can serve the society with fundamental water services, meet increasing expectations and provide new services and products, thus further contributing to a sustainable society and environment.

**Digitally transformed** end-to-end throughout the value-chain and interconnected between business units. This allows for optimal operation and efficient resource use, avoiding overconsumption.

**Predictive and proactive,** models and applications utilizing forecasts are used for control of facilities, decision support and asset management. This provides means for proactivity, sustainable decisions and efficient utilization of the systems.

**Visually communicative** with customers and society, creating customers aware of the value of water. Customers are responsible of their usage and pollution of water and discharge of used water; they accept fit-for-purpose water quality. Water is present, utilized and valued in the urban environment.

**Financially sustainable** by optimal operation (OPEX), usage, and maintenance of assets as well as sustainable investments (CAPEX). Assets and investments are planned and implemented wisely and effectively. New products and services offer new contributing revenue streams.

## 5 Key messages

• Digitalisation is a process for business development where digital solutions are used for automation and innovation.
• Water utilities are envisioned to become community orientated, digitally transformed, foresighted proactive, visually communicative and financially sustainable.
• The technical development must be based in fundamental organisational needs and improvement of services.
• Both organisational and technical developments are needed and essential to achieve effective digitalisation or digital transformation rather than digitization.
• Collection, storage, sharing and integrated analysis of large amounts of data is required to assure interoperability. This includes both soft and hard digital infrastructure.

## CRediT authorship contribution statement

**Magnus Arnell:** Conceptualization, Investigation, Writing – original draft, Project administration, Funding acquisition. **Maya Miltell:** Investigation, Writing – original draft. **Gustaf Olsson:** Writing – review & editing.

## Declaration of Competing Interest

The authors declare that they have no known competing financial interests or personal relationships that could have appeared to influence the work reported in this paper.

## Data Availability

No data was used for the research described in the article
